# From sticky to slippery: Biological and biologically-inspired adhesion and friction

**DOI:** 10.3762/bjnano.5.157

**Published:** 2014-09-03

**Authors:** Stanislav N Gorb, Kerstin Koch

**Affiliations:** 1 Functional Morphology and Biomechanics, Zoological Institute, Kiel University, Am Botanischen Garten 1–9, Kiel 24098, Germany; 2 Rhine-Waal-University of Applied Sciences, Landwehr 4, 47533 Kleve, Germany

**Figure 1:**
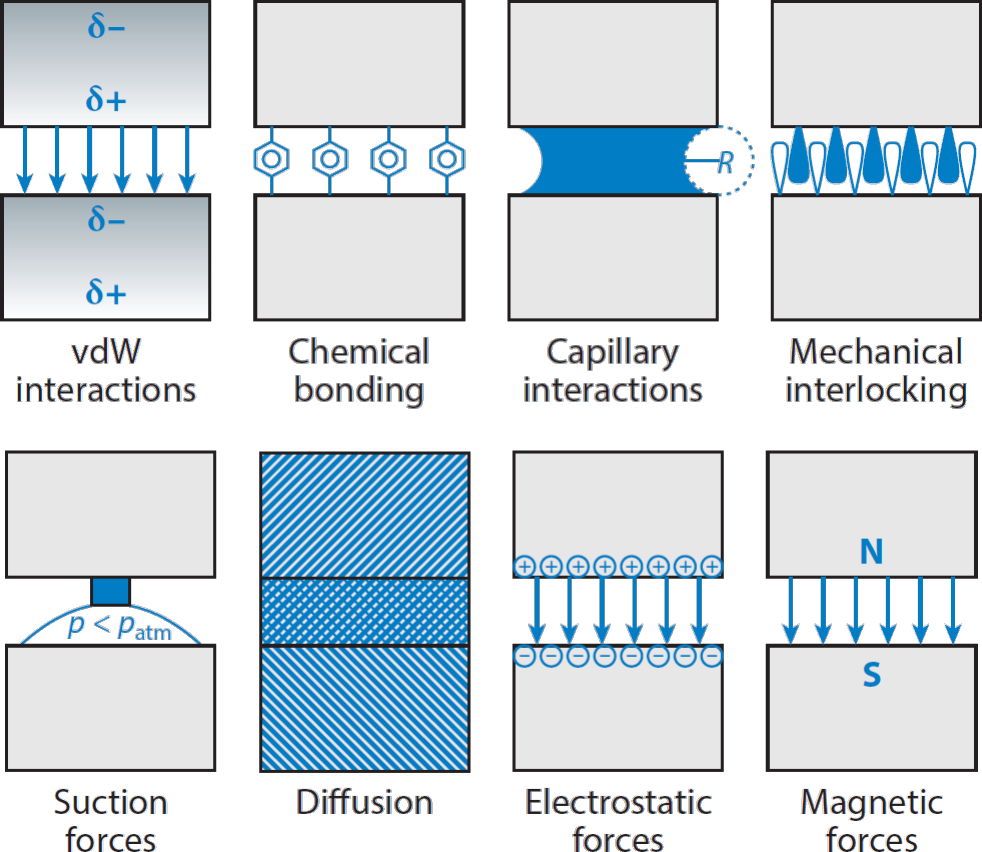
Different physical phenomena contribute to adhesion and friction in biological systems. From left to right: intermolecular van der Waals (vdW) interactions, chemical bonding, capillary interactions, mechanical interlocking, suction forces, diffusion of one surface material into the other contacting material, and electrostatic and magnetic forces. δ^+^ and δ^−^ illustrate the instantaneous formation of dipoles, *R* the curvature of the meniscus, *p* the pressure, and N and S the north pole and south pole, respectively. Reproduced with permission from [1]. Copyright 2014 Annual Reviews.

Physical phenomena such as adhesion and friction are widely-spread in biological systems. They rely on a combination of various mechanisms (Figure 1). Since living creatures move on land, in air and in water, there are numerous mechanical interactions between their body surfaces and the environment. Moreover, the motion of cells and tissues inside their bodies is an important part of developmental processes, circulation, respiration, excretion, and any other kind of motility. All these processes rely on adhesion and friction and are continuously under evolutionary pressure, which has contributed to the appearance of highly-specialized surfaces adapted to the enhancement, reduction, or optimization of their frictional and adhesive behavior. The study of these adaptations may also provide inspirations for the design of biologically-inspired artificial surfaces.

The majority of books which discuss the biomechanics of contact phenomena are restricted to selected few model systems most of which deal with materials of the human body or implants. However, a human is only one among millions of living species of organisms, and interesting adhesion- and friction-related contact problems can be found everywhere in biological systems. Different types of cells, insect feet, snake skin, plant traps, and bird wings are just a few striking examples of a tremendous diversity of biological surfaces and systems with remarkable contact behavior about many of which our knowledge is limited compared to medically relevant biotribosystems.

Since the 90s a large number of studies have been published which focus on the biotribology and the bioadhesion in various biological systems. The research on frictional and adhesive properties of very diverse biological surfaces and interfaces became a broad research field at the boundary between physics and biology. Modern experimental approaches combine a variety of microscopy methods, such as light microscopy, white-light interferometry, TEM, SEM, cryo-SEM, and AFM, with force measurement techniques at the macro-, micro- and especially at the nanoscale. This Thematic Series is a collection of experimental and theoretical studies which range from insect adhesion, bacterial adhesion and skin friction to artificial biomimetic systems, e.g., snake-skin inspired polymer patterns or gecko tape. The Thematic Series does not attempt to give a comprehensive overview of the emerging field of biological contact mechanics. Rather, it is composed of a sequence of contributions devoted to recent developments within this field. The articles highlight recent achievements in the understanding of contact phenomena in biology. They also detail the process of transferring these findings into technical materials and surfaces.

Additionally, numerous experimental methods for the characterization of the tribological properties of biological surfaces at macro-, micro-, and nanoscale levels are demonstrated in this Thematic Series. This compilation of articles is an example of interdisciplinary science as it combines approaches from biology, physics, engineering, tribology and materials science. The articles of this Thematic Series are intended to be of interest to both engineers and physicists who work with biological systems as well as to biologists who study the physics of friction and adhesion.

We would like to thank all the authors for contributing their beautiful work to this Thematic Series! Moreover, we are grateful to all referees for their promptly provided reports which facilitated the high quality of the manuscripts and also allowed us to publish in a timely manner. Finally, we thank the Editorial Office at the Beilstein-Institut for their continuous great support.

Stanislav N. Gorb and Kerstin Koch

Kiel and Kleve, July 2014
